# Supplementary material for: Automated synthetic cell-based screening for designed proteins with emergent functions
Source: Nat Commun. 2026 Aug 15;17:8415. doi: 10.1038/s41467-026-76787-8 (PMC13477476; doi:10.1038/s41467-026-76787-8)
Supplement: Supplementary file 1 [file 41467_2026_76787_MOESM1_ESM.pdf]

Supplementary information: Automated Synthetic Cell-based Screening for Designed Proteins with Emergent Functions

Kareem Al Nahas[1]*, Béla P. Frohn[1], Aleksandra Šakanović[1], Frank Siedler[1] and Petra Schwille[1]*

[1]Dept. Cellular and Molecular Biophysics, Max Planck Institute of Biochemistry; Martinsried, D-82152, Germany

*Corresponding authors

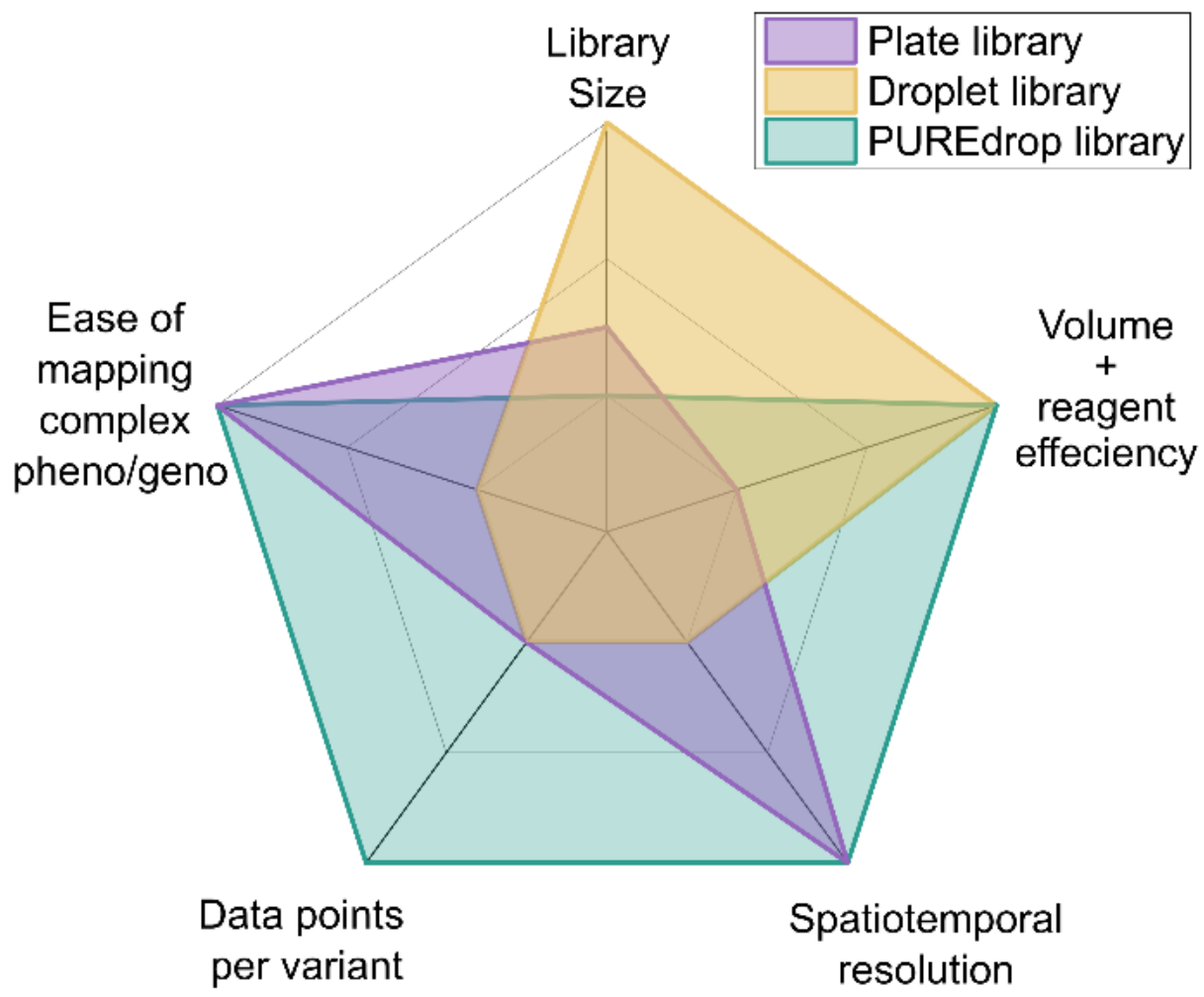

Supplementary Figure 1: Comparison between protein screening strategies grouped by library type. The schematic highlights the differences in throughput, spatiotemporal resolution, ease of extracting genotype-phenotype linkage, and data richness across bulk well plate (arrayed), droplet-based sorting (pooled), and our PUREdrop (arrayed droplets) screening pipelines.

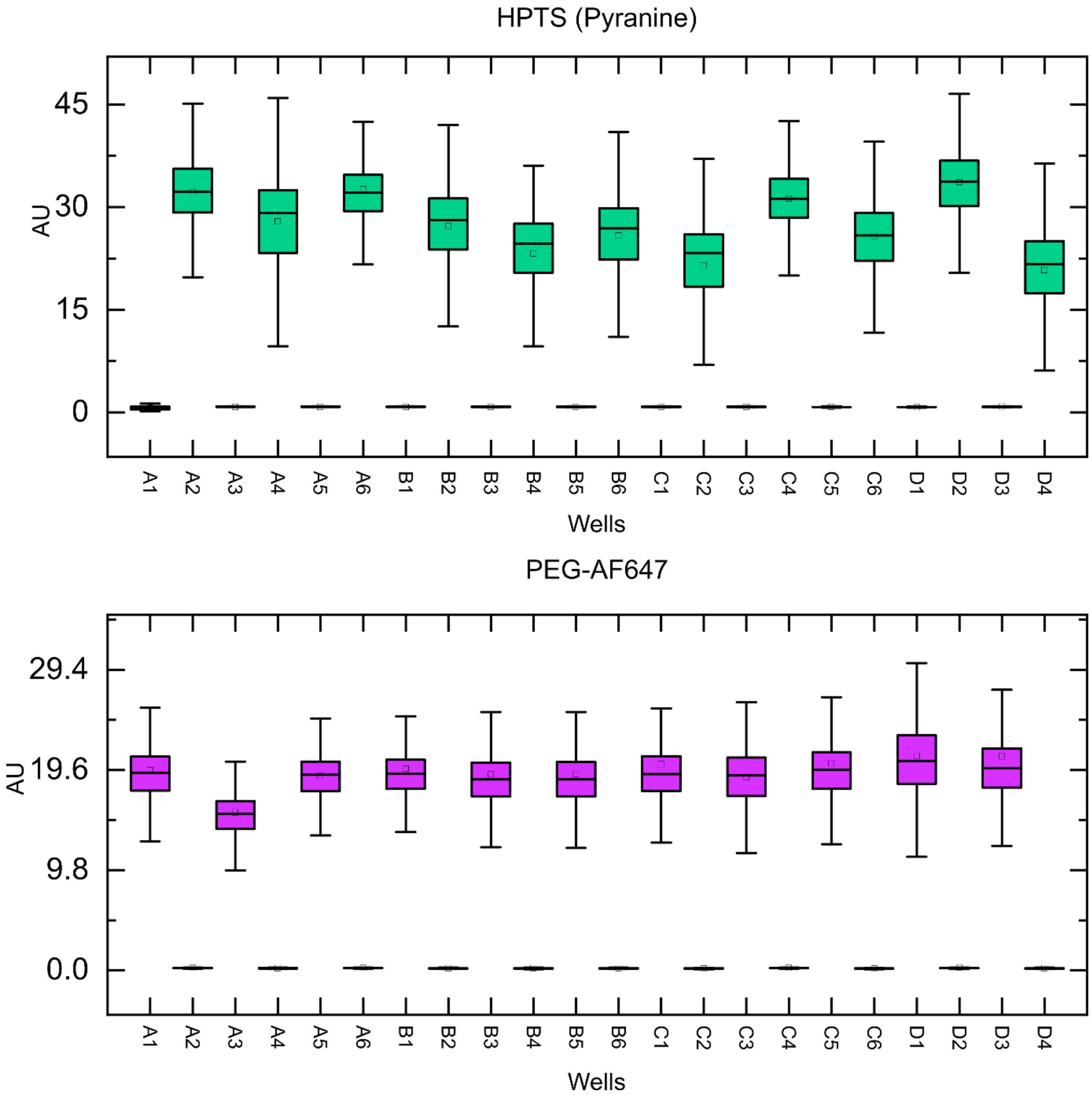

Supplementary Figure 2: Cross-contamination assay using alternating fluorescent tracers. Schematic of the alternating input-well layout and corresponding box plots of fluorescence intensity distributions for droplet populations generated from wells containing Pyranine (HPTS) or PEG-AF647. PEG-AF647 was prepared by conjugating 20 kDa PEG-azide with DBCO-AF647. Center lines indicate the median, boxes the interquartile range (IQR), and whiskers extend to 1.5 × IQR. Source data and the sample size used to derive statistics is available in the source data file.

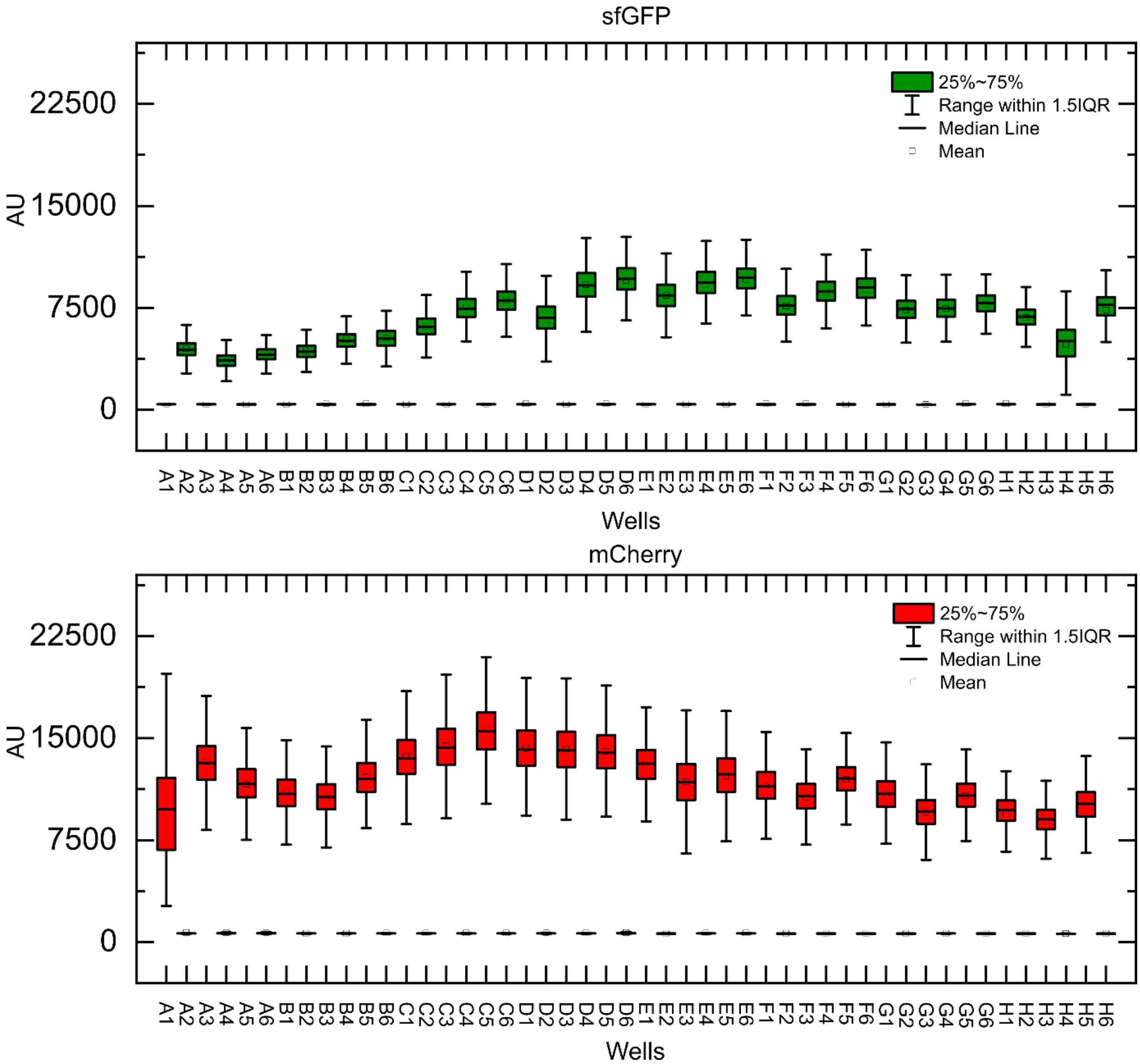

Supplementary Figure 3: Distribution of fluorescence intensities underlying Fig. 3b. Non-normalized box plots showing the distribution of fluorescence intensities measured for all imaged droplets in each well after 15 h of expression for the indicated constructs. These data correspond to the well-averaged values summarized in Fig. 3b. Center lines indicate the median, boxes the interquartile range (IQR), and whiskers extend to 1.5 × IQR. Source data and the sample size used to derive statistics is available in the source data file.

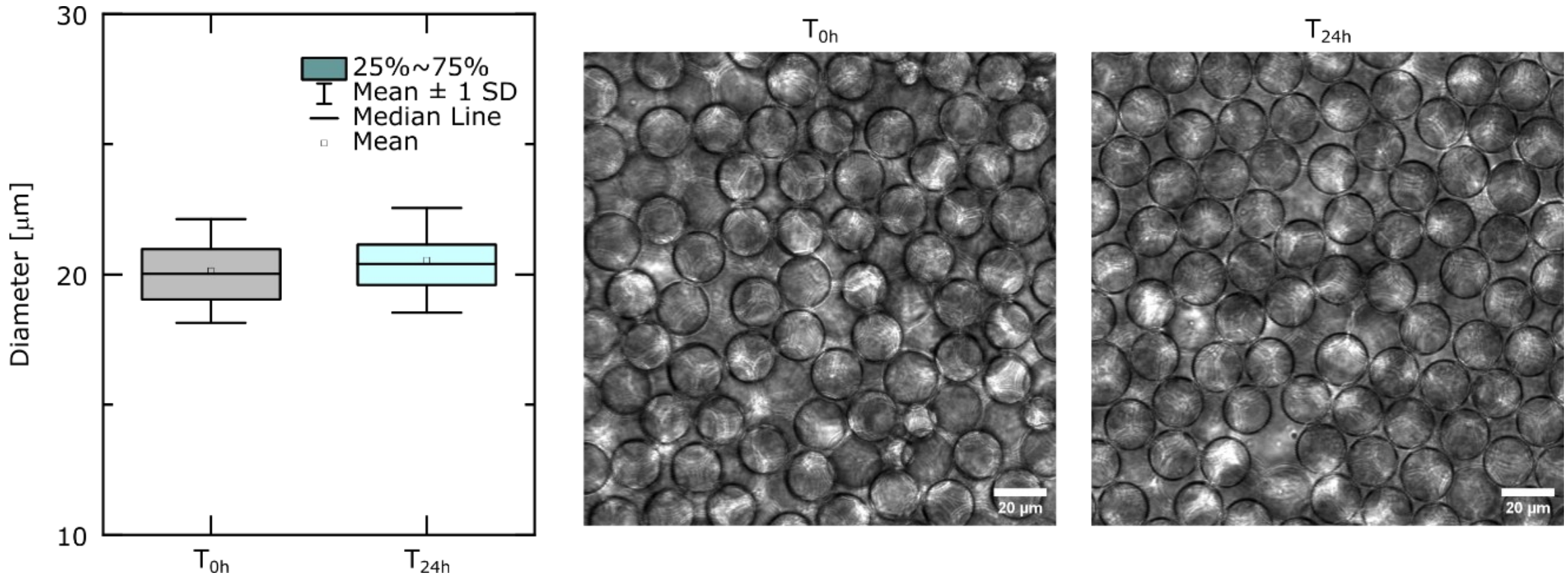

Supplementary Figure 4: Droplet stability during incubation at 30 °C.
Representative images of droplets at the start of incubation (n_0 = 31,651) and after 24 h (n_{24} = 65,535), demonstrating droplet stability over the full incubation period. Boxes indicate 25-75%, whiskers indicate mean ± SD, horizontal lines indicate median and squares indicate mean. Source data for this figure is available in the source data file.

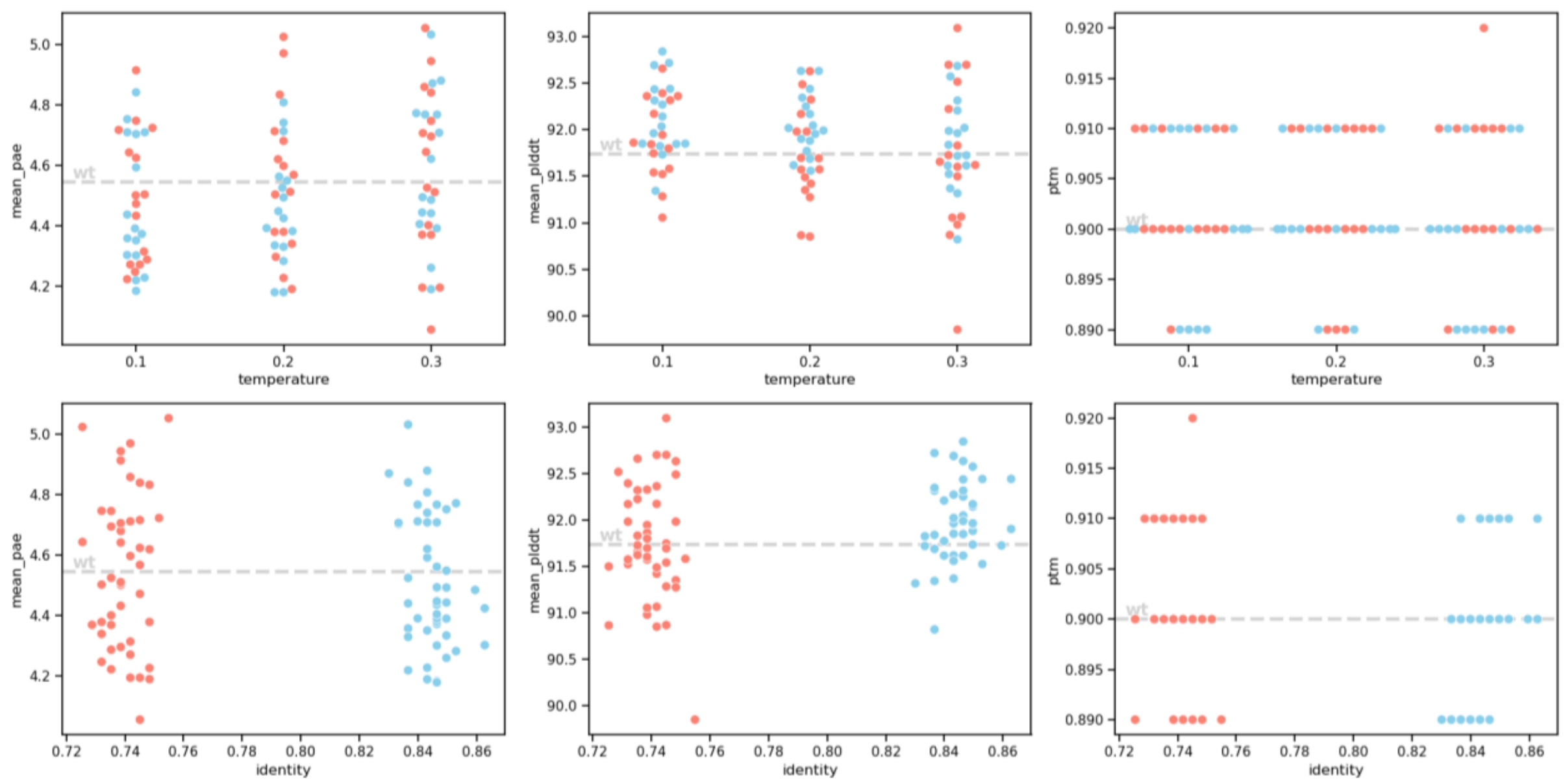

Supplementary Figure 5: Confidence metrics of ColabFold prediction of the generated sequences. 70% conservation cutoff in blue, 50% conservation cutoff in red. Source data for this figure is available in the source data file.

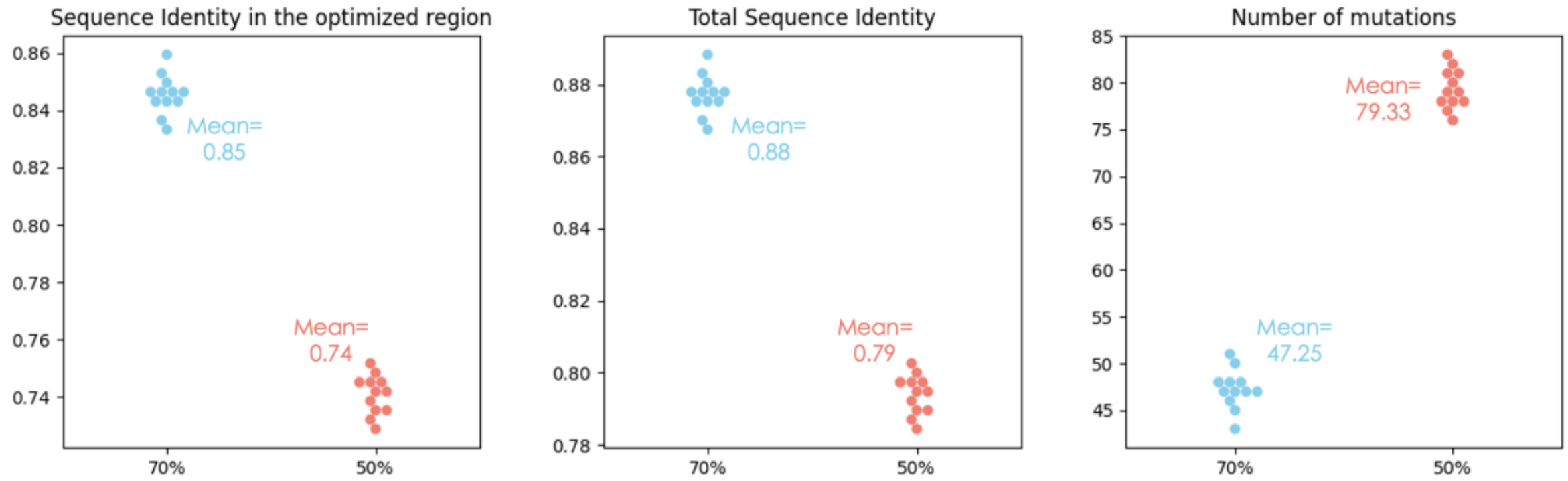

Supplementary Figure 6: Sequence conservation of the 24 tested sequences. Left: Sequence identity to *E. coli* FtsZ when only taking the optimized region into account. Middle: Sequence identity to *E. coli* FtsZ when taking the full sequence into account, including unstructured parts that are always identical. Right: Total number of mutations. Source data for this figure is available in the source data file.

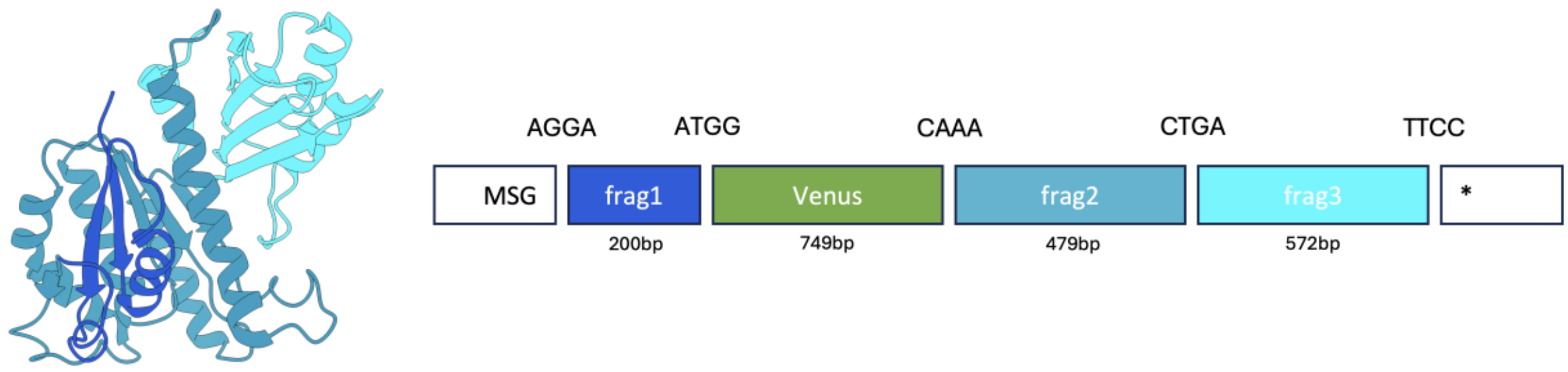

Supplementary Fig 7: Cloning scheme. All FtsZ variants and the wildtype were ordered in three fragments with the same overhangs. The fluorescent protein Venus is inserted between the first and second fragment, at position 55/56. The third fragment corresponds to the C-terminal domain.

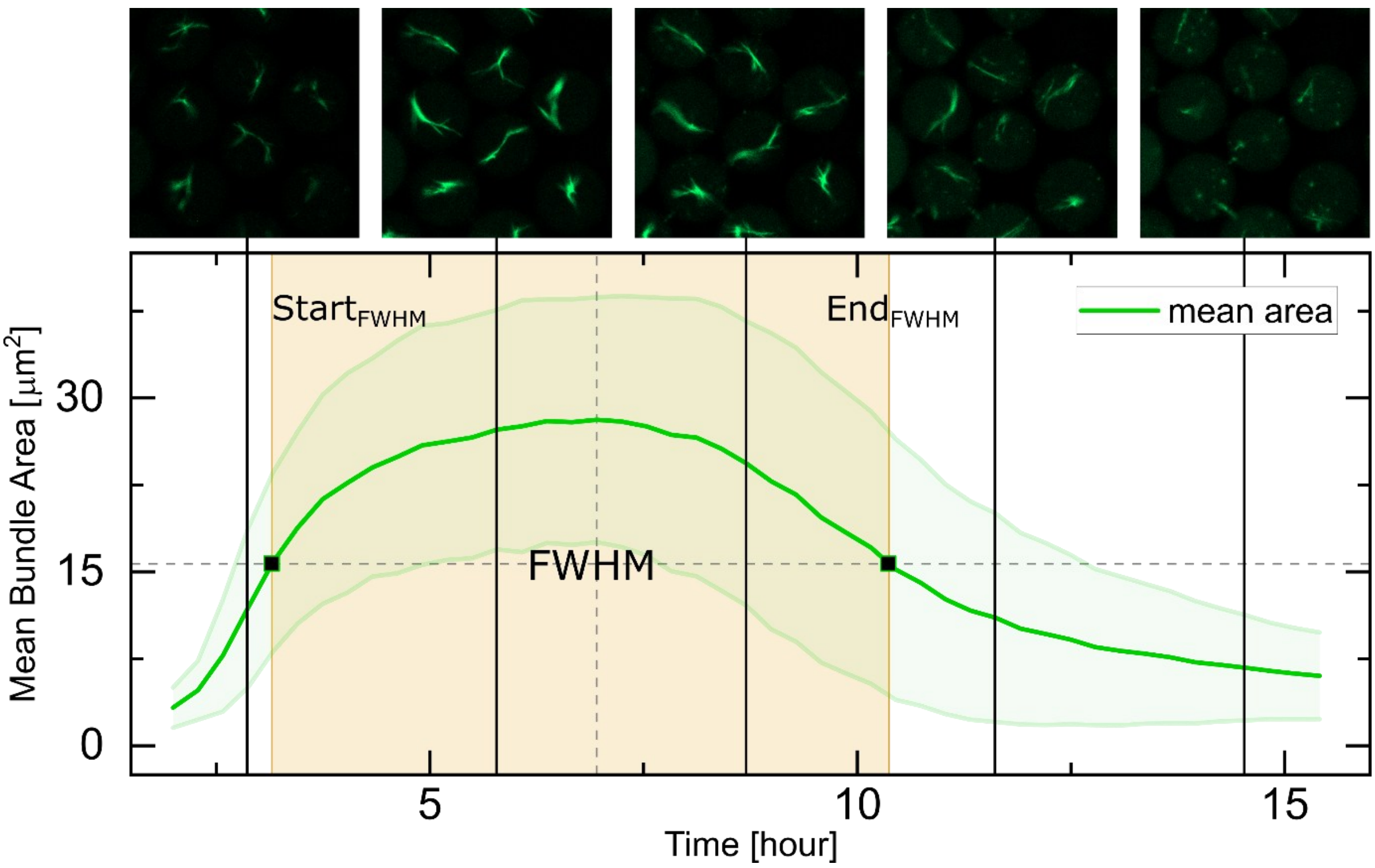

Supplementary Figure 8: Image collage of filament-bundle assembly and disassembly for the variant mpnnFtzZv18 (G5).

Representative montage showing bundle evolution at selected time points (2.9, 5.8, 8.7, 11.6 and 14.5 h), used to visually correlate changes in bundle area (FWHM) with the extracted bundle lifetimes presented in Fig. 5g.

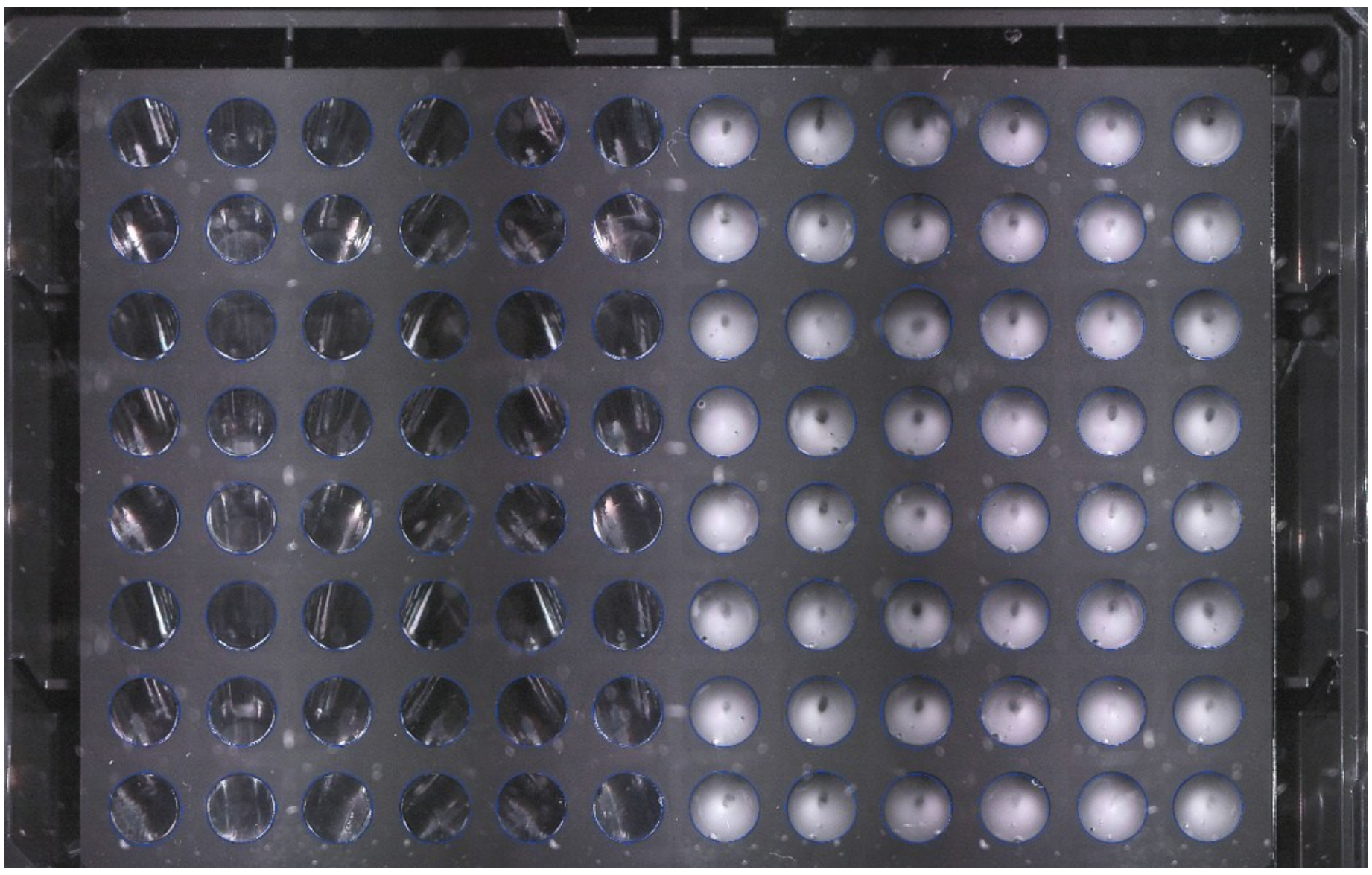

Supplementary Figure 9: Droplet packing in the output wells.

Image of the 96-well plate collected after the PUREdrop sample-preparation run, showing the uniform packing of droplets within each well. The packed droplets give each well a turbidly white appearance.

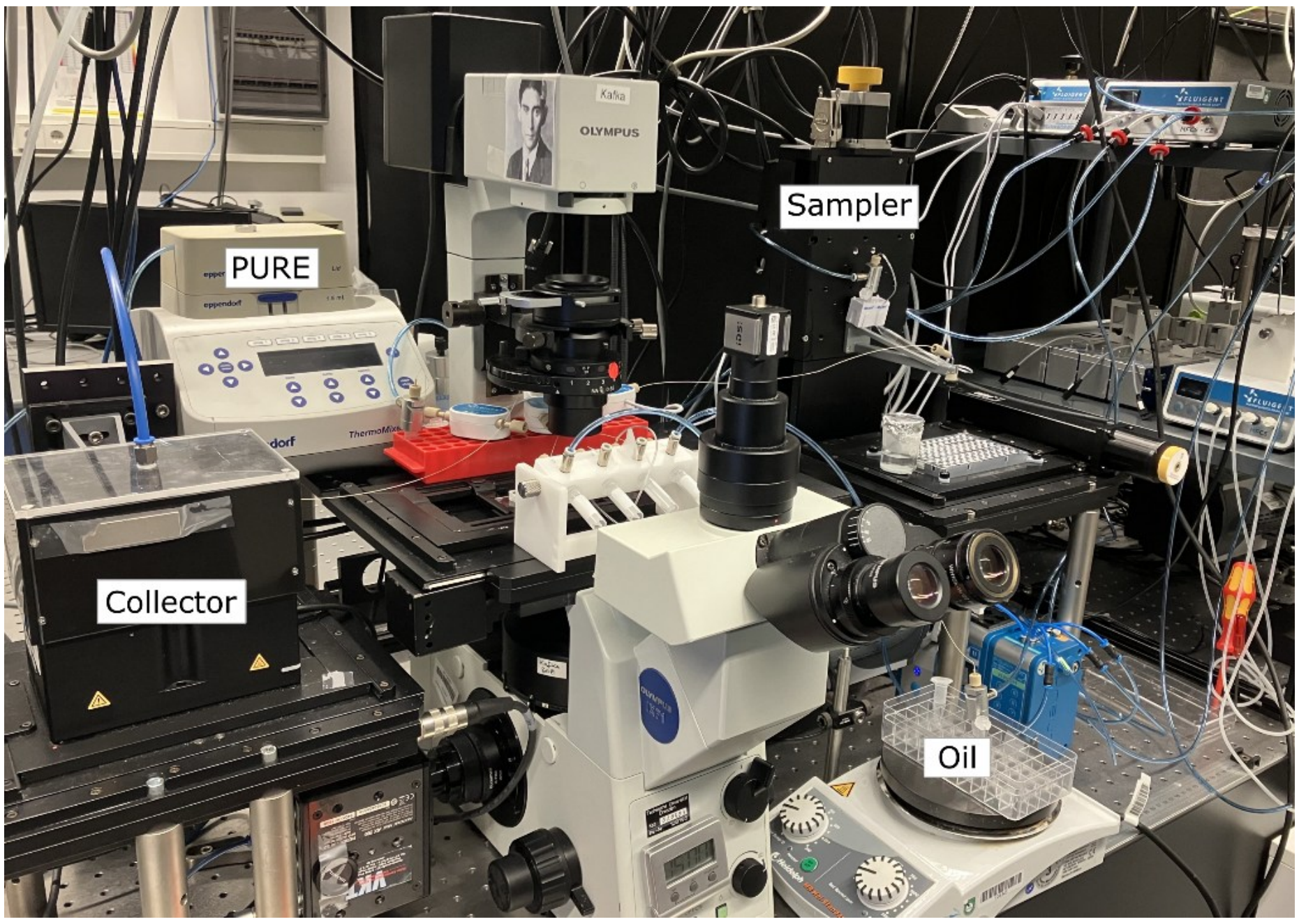

Supplementary Figure 10: Overview of the PUREdrop setup.
Image of the assembled PUREdrop system, showing the physical placement of the autosampler and fraction collector relative to the microfluidic chip, which is mounted on the microscope.

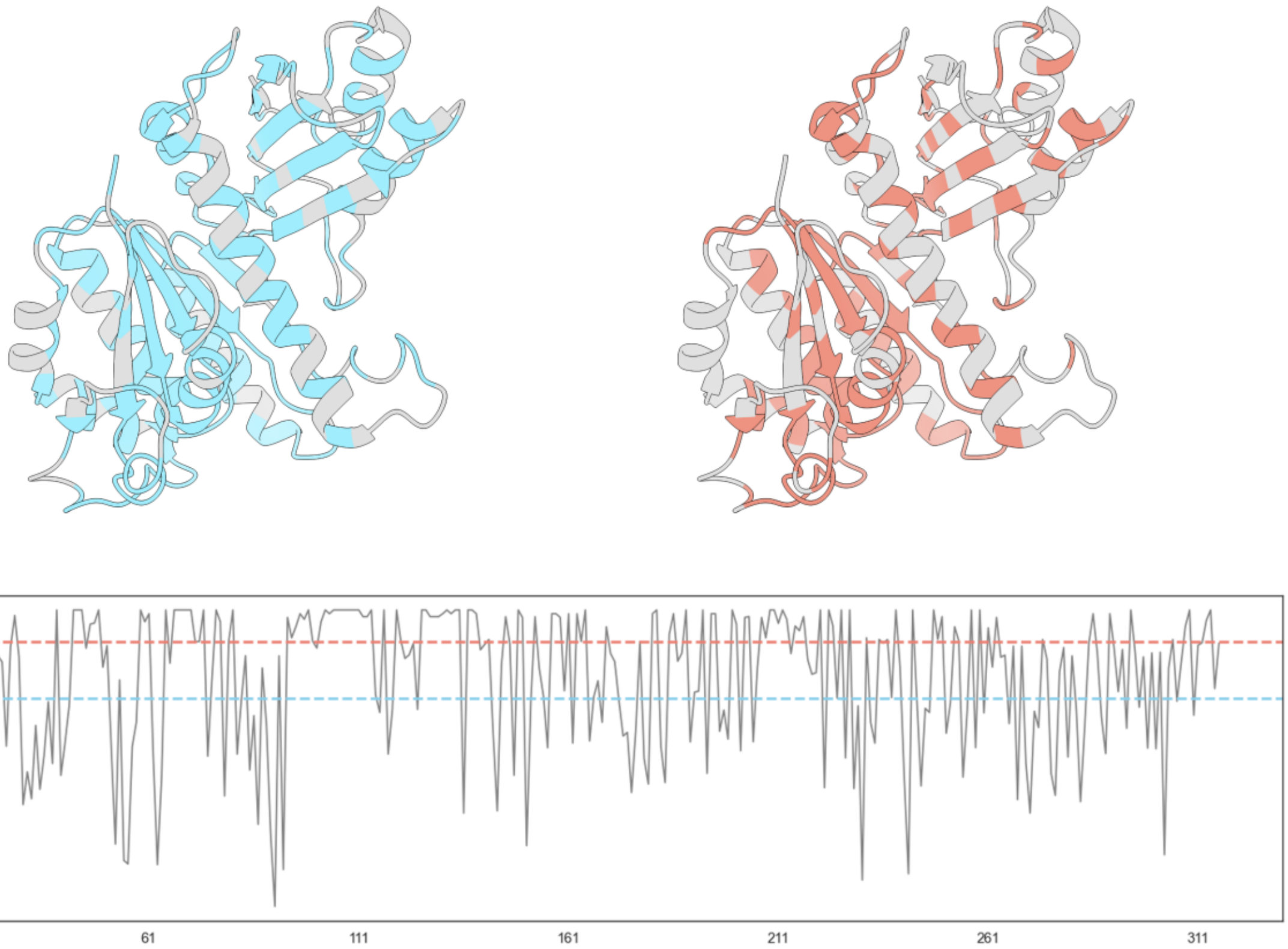

Supplementary Figure 11: Selection of conserved residues that were not re-designed. A conservation score was calculated based on entropy (lower), and the most conserved 50% (red) or 70% (blue) of the residues were fixed. Conserved residues are mostly at the active site (bottom of protein structure) and in the core. Source data for this figure is available in the source data file.

```
Kpneumoniae        1 AVIKVIGVGGGGGNAVEHMVRERIEGVEFFAVNTDAQALRKTAVGQTIQI     50
                     ||||||||||||||||||||||||||||||||||||||||||||||||||
Ecoli              1 AVIKVIGVGGGGGNAVEHMVRERIEGVEFFAVNTDAQALRKTAVGQTIQI     50

Kpneumoniae       51 GSGITKGLGAGANPEVGRNAADEDREALRAALDGADMVFIAAGMGGGTGT    100
                     |||||||||||||||||||||||||:||||||:|||||||||||||||||
Ecoli             51 GSGITKGLGAGANPEVGRNAADEDRDALRAALEGADMVFIAAGMGGGTGT    100

Kpneumoniae      101 GAAPVVAEVAKDLGILTVAVVTKPFNFEGKKRMAFAEQGITELSKHVDSL    150
                     ||||||||||||||||||||||||||||||||||||||||||||||||||
Ecoli            101 GAAPVVAEVAKDLGILTVAVVTKPFNFEGKKRMAFAEQGITELSKHVDSL    150

Kpneumoniae      151 ITIPNDKLLKVLGRGISLLDAFGAANDVLKGAVQGIAELITRPGLMNVDF    200
                     ||||||||||||||||||||||||||||||||||||||||||||||||||
Ecoli            151 ITIPNDKLLKVLGRGISLLDAFGAANDVLKGAVQGIAELITRPGLMNVDF    200

Kpneumoniae      201 ADVRTVMSEMGYAMMGSGVASGEDRAEEAAEMAISSPLLEDIDLSGARGV    250
                     ||||||||||||||||||||||||||||||||||||||||||||||||||
Ecoli            201 ADVRTVMSEMGYAMMGSGVASGEDRAEEAAEMAISSPLLEDIDLSGARGV    250

Kpneumoniae      251 LVNITAGFDLRLDEFETVGNTIRAFASDNATVVIGTSLDPDMNDELRVTV    300
                     ||||||||||||||||||||||||||||||||||||||||||||||||||
Ecoli            251 LVNITAGFDLRLDEFETVGNTIRAFASDNATVVIGTSLDPDMNDELRVTV    300

Kpneumoniae      301 VATGIG    306
                     ||||||
Ecoli            301 VATGIG    306
```

Supplementary Figure 12: Sequence Alignment of *K. pneumoniae* and *E. coli* FtsZ. The two residues that differ are not conserved and are re-designed by ProteinMPNN.

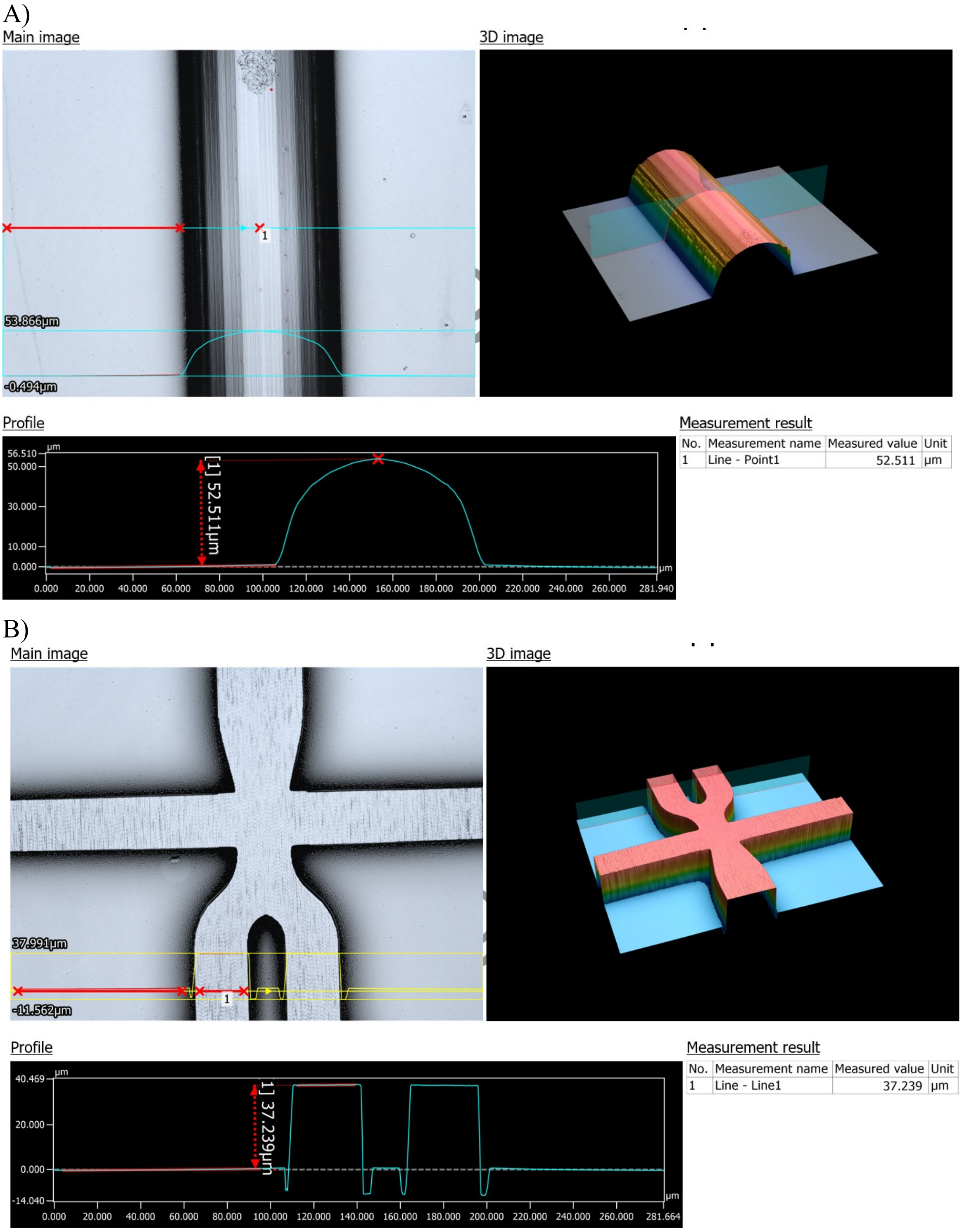

Supplementary Figure 13: Laser-profilometry measurements of the main-flow mold. a, Height map and cross-sectional profile of the rounded channel geometry used for valve operation. b Height and profile of the five-way junction region of the chip, illustrating the transitions and feature uniformity across the intersection.

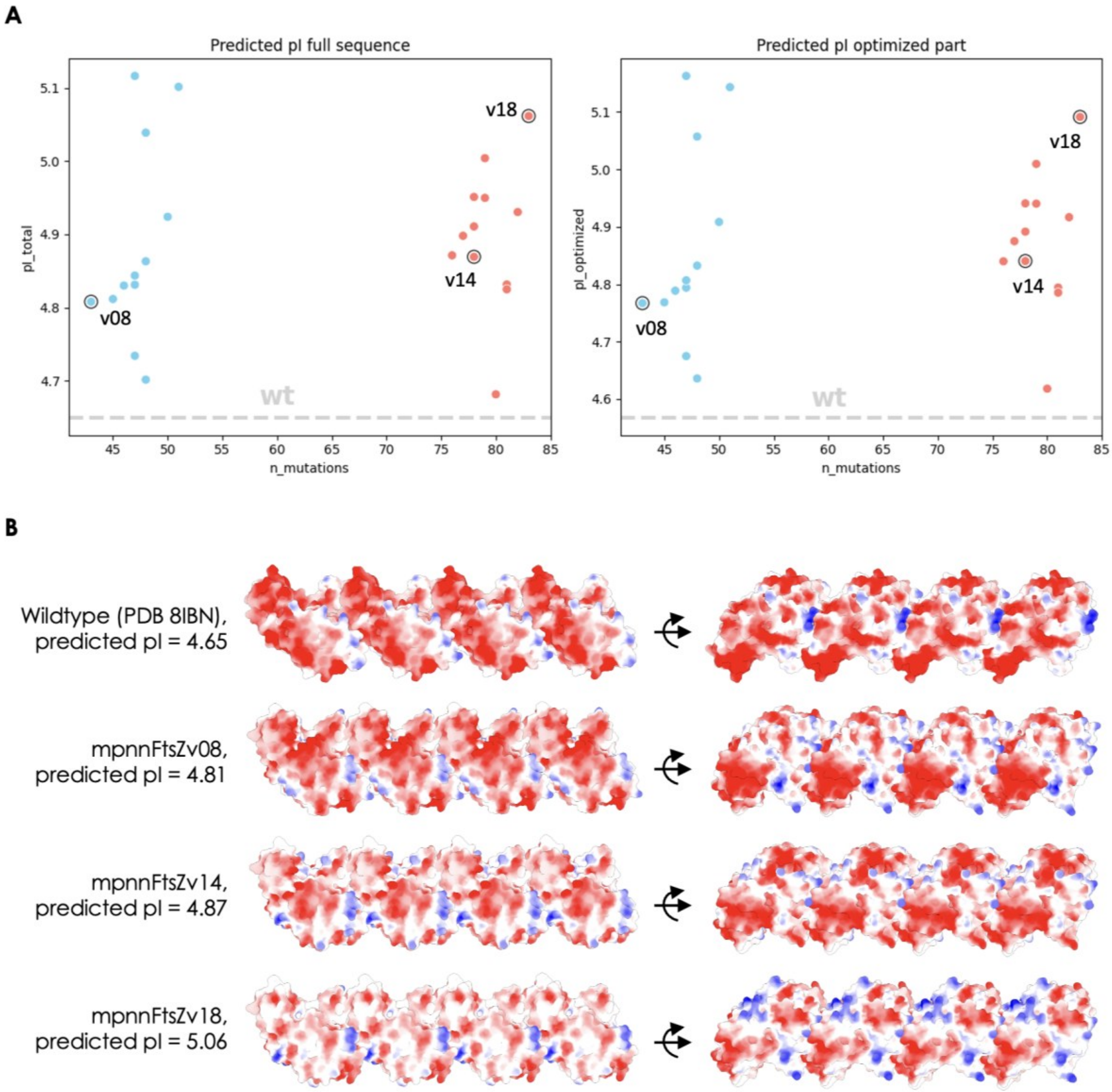

Supplementary Figure 14: Effect of ProteinMPNN re-design on surface charge. **A:** Predicted pI of re-designed FtsZ variants for full protein (left) and only globular, re-designed part (right). For all variants, ProteinMPNN redesign decreased the overall charge. 70% conservation cutoff in blue, 50% conservation cutoff in red. Variants functional in experiments highlighted with black circle. **B:** Surface colored by charge, calculated by ChimeraX. Red is negative, blue is positive. Isoelectric points were predicted with ProtParam in Biopython. While ProteinMPNN re-design reduces overall charge, it might introduce new local charged patches. Source data for this figure is available in the source data file.

Supplementary Table 1 | Sequence characteristics of FtsZ variants. Sequence ID indicates the relationship of each construct to the wild-type (WT) FtsZ. The number of mutations reflects total amino acid substitutions relative to the WT sequence. The isoelectric point (pI) represents the theoretical pH at which the protein carries no net charge (surface charge for each variant hit is depicted in Supplementary Figure 14).

Supplementary Table1: Synthetic FtsZ hits

Name	Seq_ID	n_mutations	pI_total
ecFtsZ	1.00	0	4.65
mpnnFtsZv08	0.89	43	4.81
mpnnFtsZv14	0.80	78	4.87
mpnnFtsZv18	0.78	83	5.06

Supplementary Table2: Composition of a single GGA reaction

Fragment 1 (stock 20 ng/µl)	0,075 µl
Fragment 2 (stock 40ng/µl)	0,150 µl
Fragment 3 (stock 20 ng/µl)	0,175 µl
Fragment 4 (stock 20 ng/µl)	0,200 µl
Vector (stock 666 ng/µl)	0,031 µl
H2O	0,369 µl
10x T4 Ligase Buffer (NEB M0202S)	0,200 µl
BsaI-HFv2 (NEB R3733S)	0,300 µl = 6 units / reaction
T4 Ligase (NEB M0202S)	0,500 µl = 200 units / reaction
**TOTAL**	**2 µl**

Supplementary Table3: The reaction mixture for cell lysis and FtsZ-gene variants amplification

Buffer GC (Thermo Fisher Phusion High-fidelity PCR-Kit)	10 µl
dNTPs (stock 12,5mM)	1 µl
Primer F (5'-CCCGCGAAATTAATACGACTCAC-3'; stock 50µM)	0,25 µl
Primer R (5'-CAAAAAACCCCTCAAGACCCGT-3'; stock 50µM)	0,25 µl
MgCl (stock 50mM)	2 µl
DMSO (Thermo Fisher Phusion High-fidelity PCR-Kit)	1 µl
Phusion Polymerase (stock 2 E/µl)	0,5 µl
H2O	30 µl
cells	5 µl
**TOTAL**	**50 µl**
