# Supplementary material for: Automated synthetic cell-based screening for designed proteins with emergent functions
Source: Nat Commun. 2026 Aug 15;17:8415. doi: 10.1038/s41467-026-76787-8 (PMC13477476; doi:10.1038/s41467-026-76787-8)
Supplement: Supplementary file 2 [file 41467_2026_76787_MOESM2_ESM.pdf]

Description of Additional Supplementary Files

Supplementary Data 1: Protein and DNA sequences of the wildtype, redesigned FtsZ variants and FtsZ modulators. Protein sequences are provided as a separate text file. For the FtsZ variants, the fluorescent protein Venus was inserted at G55/Q56.

Supplementary Data 2: DNA sequences of the redesigned FtsZ fragments used to assemble the variants and the ECHO pipetting scheme.

Supplementary Movie 1: Time-lapse fluorescence microscopy showing mpnnFtsZv16 (G05) bundle assembly and disassembly in cell-like compartments, corresponding to the bundle-dynamics analysis in Fig. 5.

Supplementary Movie 2: Fluorescence microscopy Z-stack of ZipA-FtsZ ring-like bundles at the interfaces between stacked droplets.

Supplementary Movie 3: Fast-camera recording of microfluidic production of PUREdroplets at ~3kHz during automated operation.
