# Supplementary material for: Automated synthetic cell-based screening for designed proteins with emergent functions
Source: Nat Commun. 2026 Aug 15;17:8415. doi: 10.1038/s41467-026-76787-8 (PMC13477476; doi:10.1038/s41467-026-76787-8)
Supplement: Supplementary file 3 [file 41467_2026_76787_MOESM8_ESM.pdf]

nature portfolio

Corresponding author(s): Petra Schwille
Kareem Al Nahas

Last updated by author(s): Jul 20, 2026

Reporting Summary

Nature Portfolio wishes to improve the reproducibility of the work that we publish. This form provides structure for consistency and transparency in reporting. For further information on Nature Portfolio policies, see our Editorial Policies and the Editorial Policy Checklist.

Statistics

For all statistical analyses, confirm that the following items are present in the figure legend, table legend, main text, or Methods section.

n/a	Confirmed	
☐	☒	The exact sample size (*n*) for each experimental group/condition, given as a discrete number and unit of measurement
☐	☒	A statement on whether measurements were taken from distinct samples or whether the same sample was measured repeatedly
☒	☐	The statistical test(s) used AND whether they are one- or two-sided<br>*Only common tests should be described solely by name; describe more complex techniques in the Methods section.*
☒	☐	A description of all covariates tested
☐	☒	A description of any assumptions or corrections, such as tests of normality and adjustment for multiple comparisons
☐	☒	A full description of the statistical parameters including central tendency (e.g. means) or other basic estimates (e.g. regression coefficient) AND variation (e.g. standard deviation) or associated estimates of uncertainty (e.g. confidence intervals)
☐	☒	For null hypothesis testing, the test statistic (e.g. *F*, *t*, *r*) with confidence intervals, effect sizes, degrees of freedom and *P* value noted<br>*Give P values as exact values whenever suitable.*
☒	☐	For Bayesian analysis, information on the choice of priors and Markov chain Monte Carlo settings
☒	☐	For hierarchical and complex designs, identification of the appropriate level for tests and full reporting of outcomes
☒	☐	Estimates of effect sizes (e.g. Cohen's *d*, Pearson's *r*), indicating how they were calculated

Our web collection on statistics for biologists contains articles on many of the points above.

Software and code

Policy information about availability of computer code

Data collection	Carl Zeiss-Zen 3.11 (blue edition), Fluigent Oxygen and SDK package, code for PUREdrop automation and computational FtsZ redesign can be found under https://github.com/KANahas/PUREdrop.git
Data analysis	ArivisPro 4.3, Python 3.11.5, OriginPro2024, Huygens Essential 25.10, UCSF ChimeraX 1.11.1), https://github.com/KANahas/PUREdrop.git

For manuscripts utilizing custom algorithms or software that are central to the research but not yet described in published literature, software must be made available to editors and reviewers. We strongly encourage code deposition in a community repository (e.g. GitHub). See the Nature Portfolio guidelines for submitting code & software for further information.

Data

Policy information about availability of data

All manuscripts must include a data availability statement. This statement should provide the following information, where applicable:

- Accession codes, unique identifiers, or web links for publicly available datasets
- A description of any restrictions on data availability
- For clinical datasets or third party data, please ensure that the statement adheres to our policy

The source data generated in this study are provided with this paper as a Source Data file. The original microscopy time-lapse datasets are available under restricted access due to their large file size (>200 GB). These data are freely available by contacting the corresponding author. Requests will normally receive an initial response within 2 weeks. Protein structures used in this work include FtsZ monomer: PDB 6UMK (https://doi.org/10.2210/pdb6UMK/pdb), FtsZ filament: PDB 8IBN

(https://doi.org/10.2210/pdb8IBN/pdb), SlmA: PDB 5HBU (https://doi.org/10.2210/pdb5hbu/pdb), ClpXP PDB 9C88 (https://doi.org/10.2210/pdb9c88/pdb), mCherry PDB 2H5Q (https://doi.org/10.2210/pdb2h5q/pdb) the remainder are predictions that can be found in the GitHub repository (https://github.com/KANahas/PUREdrop.git). The DNA sequences can be accessed in the NCBI GenBank database under accession numbers PZ544492-PZ544502 and in Source Data.

Research involving human participants, their data, or biological material

Policy information about studies with human participants or human data. See also policy information about sex, gender (identity/presentation), and sexual orientation and race, ethnicity and racism.

Reporting on sex and gender	*Use the terms sex (biological attribute) and gender (shaped by social and cultural circumstances) carefully in order to avoid confusing both terms. Indicate if findings apply to only one sex or gender; describe whether sex and gender were considered in study design; whether sex and/or gender was determined based on self-reporting or assigned and methods used. Provide in the source data disaggregated sex and gender data, where this information has been collected, and if consent has been obtained for sharing of individual-level data; provide overall numbers in this Reporting Summary. Please state if this information has not been collected. Report sex- and gender-based analyses where performed, justify reasons for lack of sex- and gender-based analysis.*
Reporting on race, ethnicity, or other socially relevant groupings	*Please specify the socially constructed or socially relevant categorization variable(s) used in your manuscript and explain why they were used. Please note that such variables should not be used as proxies for other socially constructed/relevant variables (for example, race or ethnicity should not be used as a proxy for socioeconomic status). Provide clear definitions of the relevant terms used, how they were provided (by the participants/respondents, the researchers, or third parties), and the method(s) used to classify people into the different categories (e.g. self-report, census or administrative data, social media data, etc.) Please provide details about how you controlled for confounding variables in your analyses.*
Population characteristics	*Describe the covariate-relevant population characteristics of the human research participants (e.g. age, genotypic information, past and current diagnosis and treatment categories). If you filled out the behavioural & social sciences study design questions and have nothing to add here, write "See above."*
Recruitment	*Describe how participants were recruited. Outline any potential self-selection bias or other biases that may be present and how these are likely to impact results.*
Ethics oversight	*Identify the organization(s) that approved the study protocol.*

Note that full information on the approval of the study protocol must also be provided in the manuscript.

Field-specific reporting

Please select the one below that is the best fit for your research. If you are not sure, read the appropriate sections before making your selection.

☒ Life sciences ☐ Behavioural & social sciences ☐ Ecological, evolutionary & environmental sciences

For a reference copy of the document with all sections, see nature.com/documents/nr-reporting-summary-flat.pdf

Life sciences study design

All studies must disclose on these points even when the disclosure is negative.

Sample size	No statistical method was used to predetermine sample size. Sample size was determined by the number of droplets contained within predefined imaging regions of densely packed wells. Two 400 × 400 µm tiles per well were acquired for the sfGFP-mCherry validation experiment and, unless otherwise stated, four tiles per well were acquired for the screening experiments. This yielded hundreds to thousands of droplets per condition, enabling characterization of droplet-level phenotype distributions and time-resolved dynamics.
Data exclusions	Data exclusions were performed automatically using the software-based segmentation and screening strategy described in the text. Droplets that did not meet the predefined image-analysis and quality-control criteria were excluded from the analysis.
Replication	No systematic independent replication scheme was implemented. Selected experiments and conditions were repeated in the manuscript and during method development and validation and produced consistent results. The primary analysis per sample presented was performed at the droplet level across hundreds to thousands of droplets for side-by-side comparison between repeats.
Randomization	The experiments were not randomized except in the experiment described in Figure 5. Each sample was prepared and analyzed under predefined experimental conditions.
Blinding	The experiments were not blinded except in the experiment described in Figure 5. Sample preparation, data acquisition, and analysis were automated and conducted using standardized procedures and predefined criteria, minimizing investigator bias.

Reporting for specific materials, systems and methods

We require information from authors about some types of materials, experimental systems and methods used in many studies. Here, indicate whether each material, system or method listed is relevant to your study. If you are not sure if a list item applies to your research, read the appropriate section before selecting a response.

Materials & experimental systems

n/a	Involved in the study
☒	☐ Antibodies
☒	☐ Eukaryotic cell lines
☒	☐ Palaeontology and archaeology
☒	☐ Animals and other organisms
☒	☐ Clinical data
☒	☐ Dual use research of concern
☒	☐ Plants

Methods

n/a	Involved in the study
☒	☐ ChIP-seq
☒	☐ Flow cytometry
☒	☐ MRI-based neuroimaging

Plants

Seed stocks

Report on the source of all seed stocks or other plant material used. If applicable, state the seed stock centre and catalogue number. If plant specimens were collected from the field, describe the collection location, date and sampling procedures.

Novel plant genotypes

Describe the methods by which all novel plant genotypes were produced. This includes those generated by transgenic approaches, gene editing, chemical/radiation-based mutagenesis and hybridization. For transgenic lines, describe the transformation method, the number of independent lines analyzed and the generation upon which experiments were performed. For gene-edited lines, describe the editor used, the endogenous sequence targeted for editing, the targeting guide RNA sequence (if applicable) and how the editor was applied.

Authentication

Describe any authentication procedures for each seed stock used or novel genotype generated. Describe any experiments used to assess the effect of a mutation and, where applicable, how potential secondary effects (e.g. second site T-DNA insertions, mosiacism, off-target gene editing) were examined.
