# Supplementary material for: Automated synthetic cell-based screening for designed proteins with emergent functions
Source: Nat Commun. 2026 Aug 15;17:8415. doi: 10.1038/s41467-026-76787-8 (PMC13477476; doi:10.1038/s41467-026-76787-8)
Supplement: Supplementary file 4 [file 41467_2026_76787_MOESM9_ESM.pdf]

nature portfolio

Peer Review File

Automated Synthetic Cell-based Screening for Designed Proteins with Emergent Functions

Corresponding Author: Professor Petra Schwille

This file contains all reviewer reports in order by version, followed by all author rebuttals in order by version.

Version 0:

Reviewer comments:

Reviewer #1

(Remarks to the Author)
The manuscript by Kareem Al Nahas et al. presents an automated synthetic-cell-based screening platform for designed proteins with specific functions. The platform, termed PUREdrop, is an automated system that generates droplets in 96-well plates, enabling the selection of proteins with particular functional properties from a library. PUREdrop is indeed an intriguing technology that could contribute to the design of artificial proteins. However, I found the specific objective of the present study unclear, making it difficult to assess the extent of the advance this work represents.
If the manuscript aims to highlight the versatility of the PUREdrop technology, then I believe they should demonstrate its applicability to other proteins beyond FtsZ. Conversely, if the study focuses on selecting FtsZ variants, the authors should explain more clearly why differences in FtsZ bundling or its kinetics are important for fine-tuning FtsZ function in synthetic biology. For example, do the FtsZ variants identified here result in smoother or more robust cell division in synthetic or natural cells?
Regarding FtsZ filament bundling, several proteins are known to promote the bundling. It is also established that FtsZ protofilament condensation is essential for cell division and that FtsZ treadmilling plays a crucial role. However, the present study does not appear to investigate how the selected variants relate to these key FtsZ functions associated with cell division.
Finally, if the authors intend to emphasize the broader applicability of PUREdrop to other proteins, the manuscript should include examples or use cases that illustrate how the platform could be employed in other contexts.

(Remarks on code availability)

Reviewer #2

(Remarks to the Author)
Al Nahas et al. present a well written and structured manuscript describing the development of a novel droplet-based screening method coupled to cell-free protein expression to screen designed protein variants of FtsZ. The main novelty of their system lies in automating droplet-based screening by coupling automatic liquid handling to the droplet generation device allowing different DNA templates to be tested in a defined manner. This differs from existing droplet-based screening methods in which DNA templates are introduced into droplets at single copies, leading to extremely high potential throughput, but also several caveats. The method developed here does meet a specific technological gap, namely being able efficiently characterize de novo designed proteins that need to be screened in a droplet microenvironment. What needs to be said is that the method here is filling a rather narrow niche of protein design. For a majority of protein functions (binding, enzymatic activity) the approach developed by Al Nahas is not required, and more effective cell-free based approaches with orders of magnitude higher throughput have been published. Nonetheless, the approach reported here is novel and of interest, particularly in the context of engineering synthetic cells which is also reasonably well argued by the authors in the discussion section of their manuscript. I thus recommend that the manuscript is published in Nature Communications after the authors address the specific comments below.

- Figure 1 appears to be more suitable for a review rather than an original research manuscript. The text describes the advantage / disadvantages of the various methods quite well, which is reflected in Figure 1, but the “values” in Figure 1 are

very much arbitrary. I would thus suggest to either eliminate Fig 1 entirely or at the very least relegated it to the SI.
- Although droplet-based screening and standard well-plate screening is discussed in the introduction, similarly related and relevant methods such as MITOMI based screening (for interaction screening, and enzymatic screening) should also be mentioned as it lies somewhere between droplet-based screening and the PUREdrop method described here. Furthermore, there have also been several cell-free methods recently been reported that screen interactions of designed proteins using T7 RNAP based approaches in cell-free systems (PURE).
- Fig 3b: it would be good to show the actual distribution by providing (non-normalized) box plots similar to panels g and h. At what time point was the data in b measured (pls include info in the caption).
- The authors chose to test cross-contamination by alternatively loading DNA coding for GFP and mCherry and measuring the resulting protein expression values across wells. This is a reasonable and appropriate test although maybe not the most stringent assessment because small DNA template carryovers will be masked by the larger concentration of the other template and thus not give rise to significant expression of the potentially contaminating template (loading effect). What DNA concentrations are being used here; what is the estimated final concentration per droplet? The approach described by the authors may be useful for other applications as well, where small amounts of carryover may be problematic. A more stringent test would be to simply use fluorescent dyes (linked to PEG to avoid issues with PDMS) at high concentrations, and load these alternatively into target wells, followed by quantitation to asses cross-contamination.
- The DNA assembly approach seems unnecessarily complex generating first a plasmid template, followed by transformation and then generation of a linear template. One large advantage of cell-free systems is that they work with linear templates, allowing circumvention of this entire laborious cloning process. For example, large protein libraries have been generated with PCR assembly approaches and screened in cell-free systems. Can the authors comment on their choice here in the discussion section, also in light of these other maybe more appropriate options? As a minor comment: I would have expected to find gels of the resulting DNA products for all steps of the process in the SI. What is the success rate of assembly?
- Only a total of 48 designed protein constructs were screened, which is not particularly impressive, also considering that the process is to a large extent automated. What exactly are the limitations of the system that only allowed 48 constructs to be tested? The entire screen according to Fig 5 should have been accomplished in less than one week. The authors should discuss this in the manuscript to provide the readers with an objective opinion on the limits and capabilities of the system.
- The entire manuscript is based on 48 well plates but all the figures show 96 well plates which is misleading (and related to the throughput comment above). In fact the abstract also mentions a 96 well-plate. But as far as I can tell only 48 well-plates were used?
- Regarding the resulting "hits" identified by the screen. It is not entirely clear based on the data presented what the significant differences or improvements are that were found? One argument is that the morphology of some designs differs from wild type (less branched vs more branched). But this is only supported by the handful of images provided. Can this be quantitated? A second question that arises is whether the observed differences (morphology, timing, etc) could simply be a function of different expression levels / rates between different designs arising due to differences in amino acid sequence, codon usage, etc. For example, looking at Fig 6d it appears that there is quite a difference in background fluorescence values (and hence overall protein abundance?). At a minimum the authors should take the necessary additional steps of verifying their results by expressing and purifying the constructs followed by testing them in defined and completely uniform conditions.

(Remarks on code availability)

Version 1:

Reviewer comments:

Reviewer #1

(Remarks to the Author)
In their revised manuscript, Kareem Al Nahas et al. have conducted additional experiments that effectively address my previous concerns. This experiment extends the platform from identifying functional variants within a single designed protein library to investigating emergent behavior in synthetic compartments, and I am pleased to see that these experiments further strengthen the technology. The authors have provided satisfactory responses to my questions, and I recommend the manuscript for publication.

(Remarks on code availability)

Reviewer #2

(Remarks to the Author)
The authors addressed all of my comments and strengthened the manuscript considerably through additional experiments which positions the manuscript more clearly in the synthetic cell domain.

(Remarks on code availability)

Open Access This Peer Review File is licensed under a Creative Commons Attribution 4.0 International License, which permits use, sharing, adaptation, distribution and reproduction in any medium or format, as long as you give appropriate credit to the original author(s) and the source, provide a link to the Creative Commons license, and indicate if changes were made.
In cases where reviewers are anonymous, credit should be given to 'Anonymous Referee' and the source.
The images or other third party material in this Peer Review File are included in the article's Creative Commons license, unless indicated otherwise in a credit line to the material. If material is not included in the article's Creative Commons license and your intended use is not permitted by statutory regulation or exceeds the permitted use, you will need to obtain permission directly from the copyright holder.
To view a copy of this license, visit https://creativecommons.org/licenses/by/4.0/

REPLY TO REVIEWERS

We thank the reviewers for the insightful comments and detailed review. Following their insightful suggestions, we performed additional experiments, implemented the recommended corrections, and added new explanations and clarifications throughout the manuscript. Our responses are included in this document immediately following the reviewer's comments. In the revised manuscript, the modified text is highlighted.

Reviewer #1 (Remarks to the Author):

The manuscript by Kareem Al Nahas et al. presents an automated synthetic-cell-based screening platform for designed proteins with specific functions. The platform, termed PUREdrop, is an automated system that generates droplets in 96-well plates, enabling the selection of proteins with particular functional properties from a library. PUREdrop is indeed an intriguing technology that could contribute to the design of artificial proteins. However, I found the specific objective of the present study unclear, making it difficult to assess the extent of the advance this work represents. If the manuscript aims to highlight the versatility of the PUREdrop technology, then I believe they should demonstrate its applicability to other proteins beyond FtsZ. Conversely, if the study focuses on selecting FtsZ variants, the authors should explain more clearly why differences in FtsZ bundling or its kinetics are important for fine-tuning FtsZ function in synthetic biology. For example, do the FtsZ variants identified here result in smoother or more robust cell division in synthetic or natural cells?

Regarding FtsZ filament bundling, several proteins are known to promote the bundling. It is also established that FtsZ protofilament condensation is essential for cell division and that FtsZ treadmilling plays a crucial role. However, the present study does not appear to investigate how the selected variants relate to these key FtsZ functions associated with cell division.

Finally, if the authors intend to emphasize the broader applicability of PUREdrop to other proteins, the manuscript should include examples or use cases that illustrate how the platform could be employed in other contexts.

We thank the reviewer for this important comment. We appreciate that the original version of the manuscript did not sufficiently distinguish whether the main advance was the identification of functional FtsZ variants from a computationally designed library or the broader capability of the PUREdrop platform. In response, we have significantly revised the manuscript to clarify that the primary contribution of this work is the development and validation of PUREdrop as a generalizable screening platform for emergent protein function in synthetic cells, with high synergy for screening computationally designed libraries.

To directly address this point, and in line with the editor's guidance, we added a second showcase study demonstrating platform generalizability beyond screening a redesigned FtsZ library alone. In this whole new section, we used PUREdrop to screen a functionally diverse panel of FtsZ-modulating proteins and peptides, including canonical positive and negative regulators, as well as perturbative factors acting through distinct mechanisms. This experiment extends the platform from identifying functional variants within a single designed protein library to interrogating multi-component emergent behavior in synthetic compartments. Because each candidate was co-expressed individually with fluorescently labelled FtsZ, the resulting phenotypes could be quantified through changes in FtsZ bundle morphology, persistence, and spatial localization. Importantly, this screen revealed a broad spectrum of distinct outcomes, including shorter-lived assemblies, persistent compact bundles, and, most notably, ZipA-dependent interface-localized ring-like bundle morphologies, which were not observed in the original variant screen. We believe these new data substantially strengthen the manuscript by demonstrating that PUREdrop can be applied not only to redesigned variants of one protein, but also to the screening of interacting factors that shape higher-order cytoskeletal

organization. This addition better positions the manuscript as a platform paper with clear relevance to both protein engineering and synthetic cell reconstitution.

The new second showcase can be found in the Main section under the section: "Screening of emergent function modulators", ***pages 10-12***

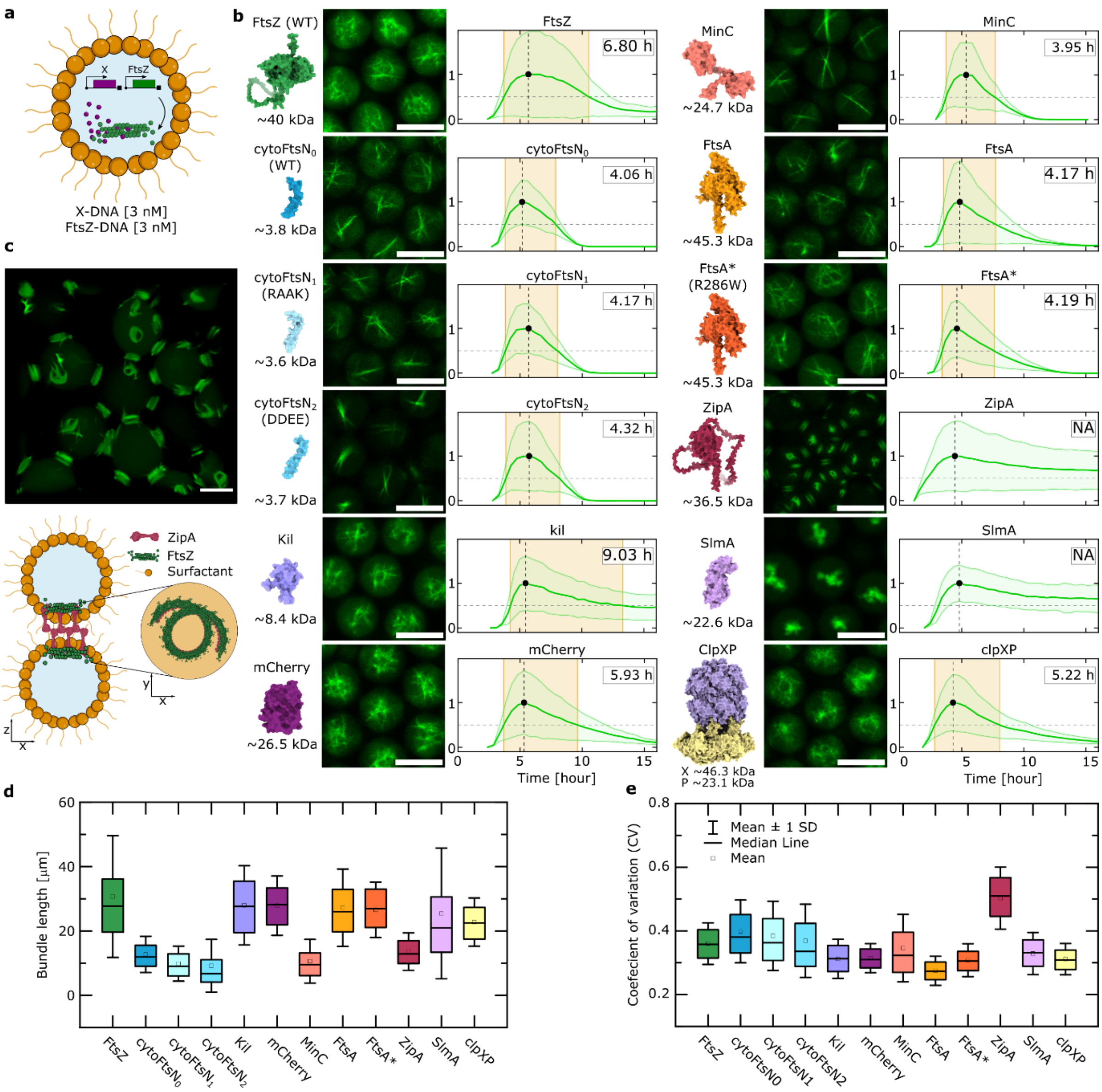

Revision Fig. 1: Screening of emergent function modulators. a Schematic of co-encapsulation of genes encoding FtsZ and the modulator of interest at similar concentrations (final 3 nM). **b** Structure and size of each protein shown alongside representative fluorescence microscopy images at 5.7 h and corresponding temporal profiles of normalized mean bundle area per droplet. Green shading indicates standard deviation. The yellow region denotes the full width at half maximum (FWHM) of the assembly-disassembly curve. Where applicable, the FWHM value is indicated as the lifetime (h). Scale bars represent 20 µm. Protein structure visualizations were prepared using UCSF ChimeraX **c** Representative deconvolved z-stack image of a sample expressing ZipA, revealing the three-dimensional arrangement of droplets, with a schematic illustrating localization of ring-like FtsZ bundles at the interface between vertically stacked droplets. **d, e** Box plots comparing bundle length and coefficient of variation

(CV) of signal intensity for skeletonized bundles per droplet at 5.7 h across all FtsZ-modulator combinations FtsZ, $CytoFtsN_0$, $CytoFtsN_1$, $CytoFtsN_2$, Kil, mCherry, MinC, FtsA, FtsA*, ZipA, SlmA, and ClpXP, with corresponding N values of 730, 1446, 1323, 1011, 1038, 1156, 1100, 777, 1184, 1011, 813, and 1459, respectively.

Reviewer #2 (Remarks to the Author):

Al Nahas et al. present a well written and structured manuscript describing the development of a novel droplet-based screening method coupled to cell-free protein expression to screen designed protein variants of FtsZ. The main novelty of their system lies in automating droplet-based screening by coupling automatic liquid handling to the droplet generation device allowing different DNA templates to be tested in a defined manner. This differs from existing droplet-based screening methods in which DNA templates are introduced into droplets at single copies, leading to extremely high potential throughput, but also several caveats. The method developed here does meet a specific technological gap, namely being able efficiently characterize de novo designed proteins that need to be screened in a droplet microenvironment. What needs to be said is that the method here is filling a rather narrow niche of protein design. For a majority of protein functions (binding, enzymatic activity) the approach developed by Al Nahas is not required, and more effective cell-free based approaches with orders of magnitude higher throughput have been published. Nonetheless, the approach reported here is novel and of interest, particularly in the context of engineering synthetic cells which is also reasonably well argued by the authors in the discussion section of their manuscript. I thus recommend that the manuscript is published in Nature Communications after the authors address the specific comments below.

1. Figure 1 appears to be more suitable for a review rather than an original research manuscript. The text describes the advantage / disadvantages of the various methods quite well, which is reflected in Figure 1, but the "values" in Figure 1 are very much arbitrary. I would thus suggest to either eliminate Fig 1 entirely or at the very least relegated it to the SI.

We thank the reviewer for this suggestion and agree that the conceptual comparison shown in Fig. 1 was intended mainly to help readers visualize the methodological gap addressed by PUREdrop, but is more appropriate as supporting material than as a main figure. We have therefore **moved Fig. 1 to the Supplementary Information** (now **Supplementary Fig. 1**), revised the main text accordingly, and renumbered the subsequent figures.

2. Although droplet-based screening and standard well-plate screening is discussed in the introduction, similarly related and relevant methods such as MITOMI based screening (for interaction screening, and enzymatic screening) should also be mentioned as it lies somewhere between droplet-based screening and the PUREdrop method described here. Furthermore, there have also been several cell-free methods recently been reported that screen interactions of designed proteins using T7 RNAP based approaches in cell-free systems (PURE).

We thank the reviewer for this suggestion. We agree that, although some of these approaches were already cited, their relevance especially that of **MITOMI-based screening** was not stated explicitly enough. We have therefore revised the Introduction to position PUREdrop more clearly relative to these intermediate microfluidic methods and added an additional relevant reference.

The revised text *can be found in the **Introduction** section, **page 2***

»This exposes a gap in screening methods for emergent function phenotypes (Supplementary Fig. 1), and underscores the need for intermediate approaches that bridge bulk well-plate and droplet-based

technologies similar to the MITOMI-based screening approaches [27–29], offering the spatiotemporal resolution required, while combining the genotype-phenotype mapping of microtiter plates with the statistical power and efficiency of droplet assays.«

References used describing cell-free screening methods (18- 21, 25-29)

MITOMI references:

27. Markin, C. J. et al. Revealing enzyme functional architecture via high-throughput microfluidic enzyme kinetics. Science (1979). 373, (2021).

28. Swank, Z., Laohakunakorn, N. & Maerkl, S. J. Cell-free gene-regulatory network engineering with synthetic transcription factors. Proc. Natl. Acad. Sci. U. S. A. 116, 5892–5901 (2019).

29. Baranwal, A. K. & Maerkl, S. J. Microfluidics meets cell-free systems: from molecular engineering to synthetic cells. Curr. Opin. Biotechnol. 96, 103384 (2025).

3. Fig 3b: it would be good to show the actual distribution by providing (non-normalized) box plots similar to panels g and h. At what time point was the data in b measured (pls include info in the caption).

We thank the reviewer for this suggestion. We have now clarified in the caption that the values shown in Fig. 3b correspond to measurements taken after 15 hours.
»Heat maps showing the average fluorescence intensity of all imaged droplets per well (after 15 hours).«
In addition, we generated the requested non-normalized box plots to show the underlying intensity distributions and included them in the Supplementary Information (Supplementary Fig. 3).

4. The authors chose to test cross-contamination by alternatively loading DNA coding for GFP and mCherry and measuring the resulting protein expression values across wells. This is a reasonable and appropriate test although maybe not the most stringent assessment because small DNA template carryovers will be masked by the larger concentration of the other template and thus not give rise to significant expression of the potentially contaminating template (loading effect). What DNA concentrations are being used here; what is the estimated final concentration per droplet? The approach described by the authors may be useful for other applications as well, where small amounts of carryover may be problematic. A more stringent test would be to simply use fluorescent dyes (linked to PEG to avoid issues with PDMS) at high concentrations, and load these alternatively into target wells, followed by quantitation to asses cross-contamination.

We thank the reviewer for this suggestion. We have now added the DNA concentrations to the figure caption (9 nM in the input plate, corresponding to 3 nM after encapsulation). In addition, we performed the suggested carryover experiment using alternating wells containing Pyranine (HPTS) and PEG-AF647 (prepared by click conjugation of 20 kDa PEG-azide with DBCO-AF647), and included the resulting data in the Supplementary Information (Supplementary Fig. 2). The new experiment showed similarly minimal carryover between alternating wells.

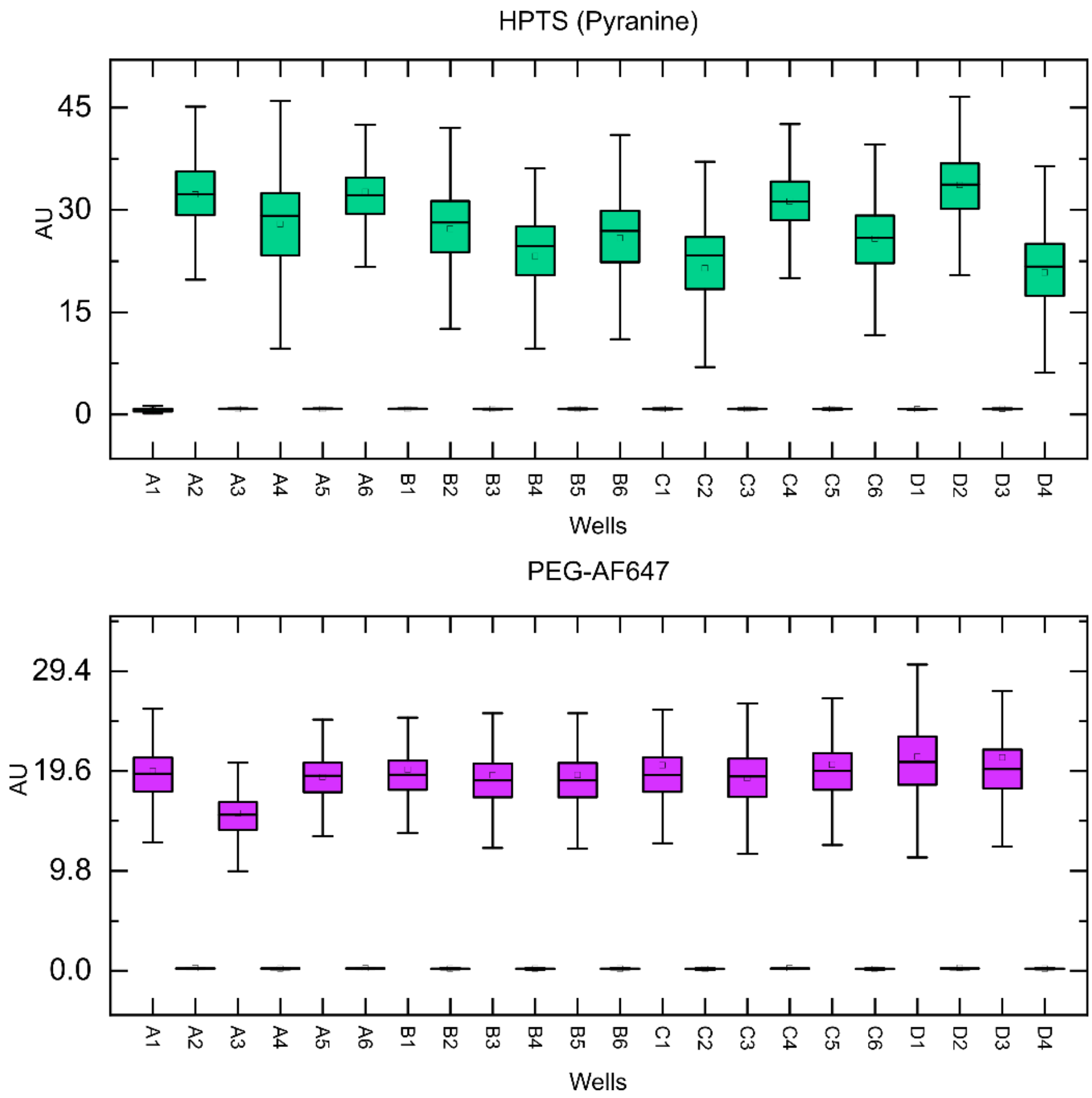

Revision Fig. 2: Cross-contamination assay using alternating fluorescent tracers. Box plots showing fluorescence intensity distributions for droplet populations generated from alternating input wells containing Pyranine (HPTS) or PEG-AF647. PEG-AF647 was prepared by conjugating 20 kDa PEG-azide with DBCO-AF647. Center lines indicate the median, boxes the interquartile range (IQR), and whiskers extend to 1.5 × IQR.

5. The DNA assembly approach seems unnecessarily complex generating first a plasmid template, followed by transformation and then generation of a linear template. One large advantage of cell-free systems is that they work with linear templates, allowing circumvention of this entire laborious cloning process. For example, large protein libraries have been generated with PCR assembly approaches and screened in cell-free systems. Can the authors comment on their choice here in the discussion section, also in light of these other maybe more appropriate options? As a minor comment: I would have expected to find gels of the resulting DNA products for all steps of the process in the SI. What is the success rate of assembly?

We thank the reviewer for this comment. We agree that cell-free systems are compatible with direct linear DNA templates and that PCR-based assembly can provide a faster route from design to screening. In the revised manuscript, we now state this explicitly and clarify that we chose a plasmid-based intermediate for robustness and because it interfaces well with our existing DNA-handling workflow. We also now note that users can bypass cloning and transformation altogether and use direct PCR-based library assembly for an even faster design-to-screen cycle.

*In the **Main** section, **page 6***

»To prepare screening-ready libraries within a few days, we integrated PUREdrop with a quick DNA library assembly protocol (Fig. 4). The process begins with gene fragments containing Golden Gate Assembly (GGA) overhangs, delivered in plates compatible with the ECHO acoustic liquid handler. Using the ECHO, 2 µl GGA reactions containing vector, restriction enzyme, ligase, buffer and selected gene fragments are assembled and incubated at 37 °C for 3 h, after which the products are transformed into chemically competent cells and grown overnight. This part of the workflow builds on and is fully compatible with the semi-automated protein production (SAPP) pipeline [4]. PURE-compatible DNA templates, comprising a T7 promoter, coding sequence and T7 terminator, are then generated directly from bacterial lysate by PCR amplification. In practice, this allows users following the SAPP workflow to use a small aliquot of the same overnight culture prepared for protein production to also generate templates for PUREdrop, without requiring additional steps. While we implemented a plasmid-based intermediate for robustness and straightforward integration with SAPP, users can also exploit the compatibility of cell-free expression with linear DNA templates to bypass cloning and transformation altogether, enabling an even faster design-to-screen cycle through direct PCR-based library assembly. «

Regarding gel validation and assembly success rate, our workflow largely follows the SAPP protocol by Qian et al. [15], where genes of comparable complexity where assembled for usage in subsequent purification. Their resulting purified proteins showed the expected mass in 90% of the cases, which is a very conservative lower limit of assembly success rate. As not all their proteins might have been expressed and successfully purified, assembly success rate is likely close to perfect. We hence did not systematically characterize each intermediate by gel electrophoresis, as our workflow was developed as a rapid screening pipeline rather than a stepwise DNA optimization protocol.

15. Qian, J. et al. Accelerating Protein Design by Scaling Experimental Characterization. bioRxiv http://biorxiv.org/lookup/doi/10.1101/2025.08.05.668824 (2025).

6. Only a total of 48 designed protein constructs were screened, which is not particularly impressive, also considering that the process is to a large extent automated. What exactly are the limitations of the system that only allowed 48 constructs to be tested? The entire screen according to Fig 5 should have been accomplished in less than one week. The authors should discuss this in the manuscript to provide the readers with an objective opinion on the limits and capabilities of the system. The entire manuscript is based on 48 well plates but all the figures show 96 well plates which is misleading (and related to the throughput comment above). In fact the abstract also mentions a 96 well-plate. But as far as I can tell only 48 well-plates were used?

We agree that the manuscript should distinguish more clearly between the current operating format and the platform's potential throughput. In the present study, we used 96-well plates throughout, but populated 48 wells per run. We have revised the manuscript to make this explicit and to avoid confusion arising from the plate format shown in the figures. We also now clarify in the Methods that this choice reflected a balance between preparation time and downstream time-lapse imaging with sufficient temporal resolution, rather than a fundamental limit of the platform. In addition, we expanded the description of the operational loop and highlighted clear opportunities for runtime optimization that could enable 96-well preparation within a similar timeframe.

*In the **Methods** section, **page 15** **(Lines 12-23)***

»The operational loop requires 375 seconds per well and begins with the washing step (140 s), followed by priming (35 s) and then droplet production (100 s). Throughout all three stages, oil is continuously perfused through the chip serving multiple important functions. In addition to its essential role in droplet generation, the oil clears residual droplets from the output tubing and forms a stabilizing barrier that prevents evaporation during overnight measurements. In the present study, although droplets were sorted into a 96-well plate format, only 48 wells were populated per run to balance preparation time with downstream time-lapse imaging and temporal resolution. There is considerable room for optimization to reduce the total runtime and scale up to 96-well preparation within the same timeframe. For instance, increasing the flow rates during the washing steps, refining mechanical movements (58 s) and reducing the production time could cut those times in half, ultimately enabling faster and larger-scale library preparation. «

7. Regarding the resulting "hits" identified by the screen. It is not entirely clear based on the data presented what the significant differences or improvements are that were found? One argument is that the morphology of some designs differs from wild type (less branched vs more branched). But this is only supported by the handful of images provided. Can this be quantitated?

We agree that morphology should be supported quantitatively rather than primarily by representative images. In the revised manuscript, we now quantify morphology in both screening case studies using skeletonized segmented bundles and two complementary metrics: skeleton length, which reports the spatial extent of the assemblies, and the coefficient of variation (CV) of fluorescence intensity, used as an image-based proxy for filament bundling. In addition, we revised the corresponding figure subpanels (Fig. 5) to include box plots comparing these metrics across conditions. Together, these changes provide a quantitative basis for distinguishing more extended, network-like assemblies from shorter, more compact bundled structures, rather than relying on representative images alone.

*In the **Main** section, **page 9-10** **(Lines 39-41)***

» To quantify bundle morphology across the selected hit variants, we analyzed skeletonized bundles at 7.3 h using two complementary metrics. The first is skeleton length, reflecting the spatial extent of the assemblies, and the other is the coefficient of variation (CV) of fluorescence intensity, an image-based proxy for filament bundling [37].«

37. Higaki, T., Akita, K. & Katoh, K. Coefficient of variation as an image-intensity metric for cytoskeleton bundling. Sci. Rep. 10, 22187 (2020).

8. A second question that arises is whether the observed differences (morphology, timing, etc) could simply be a function of different expression levels / rates between different designs arising due to differences in amino acid sequence, codon usage, etc. For example, looking at Fig 6d it appears that there is quite a difference in background fluorescence values (and hence overall protein abundance?). At a minimum the authors should take the necessary additional steps of verifying their results by expressing and purifying the constructs followed by testing them in defined and completely uniform conditions.

We thank the reviewer for this important comment. We agree that sequence-dependent differences in expression level and expression kinetics can contribute to the phenotypes observed in a cell-free screening assay. In our view, however, this is also part of the value of

the PUREdrop readout: the platform is designed to capture the combined genotype-to-phenotype behavior of each construct in a confined TXTL environment, where expression, assembly and higher-order function are inherently coupled. For synthetic-cell applications, this integrated readout is relevant because the usefulness of a construct depends not only on its intrinsic biophysical properties, but also on how effectively it can be produced and self-organize under compartmentalized, resource-limited conditions.

At the same time, we agree that follow-up characterization under purified and fully controlled conditions is important to disentangle expression effects from intrinsic assembly differences. However, we see this as the natural next step after the screen, but beyond the scope of the present manuscript, whose primary aim is to establish PUREdrop as a platform for screening emergent phenotypes directly from DNA in synthetic cells.

REPLY TO REVIEWERS

Reviewer #1

In their revised manuscript, Kareem Al Nahas et al. have conducted additional experiments that effectively address my previous concerns. This experiment extends the platform from identifying functional variants within a single designed protein library to investigating emergent behavior in synthetic compartments, and I am pleased to see that these experiments further strengthen the technology. The authors have provided satisfactory responses to my questions, and I recommend the manuscript for publication.

Reviewer #2

The authors addressed all of my comments and strengthened the manuscript considerably through additional experiments which positions the manuscript more clearly in the synthetic cell domain.

We thank both reviewers for their positive assessment of the revised manuscript. We are pleased that the additional experiments and revisions addressed their previous concerns, strengthened the study, and clarified its relevance to the synthetic cell field. We greatly appreciate their constructive feedback and recommendation for publication.
